# Chronic locked posterior gleno-humeral dislocation: technical note on fibular grafting for restoration of humeral head sphericity

**DOI:** 10.1186/s13018-021-02835-2

**Published:** 2021-11-18

**Authors:** Amr Abdel-Mordy Kandeel

**Affiliations:** Department of Orthopedics and Traumatology, Faculty of Medicine, Menoufia University, Gamal Abdel-Nasser Street, Shebien El-kom, Menoufia Governorate Egypt

**Keywords:** Fibular grafting, Humeral head reconstruction, Locked gleno-humeral dislocation, Posterior gleno-humeral dislocation, Reverse Hill-Sachs defect

## Abstract

**Background:**

Reconstruction of reverse Hill-Sachs defect using osteo-chondral allograft has the advantages of spherical re-contouring and provision of smooth biological articular surface of the reconstructed humeral head. However, worldwide availability and risk of disease transmission of osteo-chondral allograft remain points of increasing concerns. As an alternative to lacking osteo-chondral allograft, the current technical note describes a reconstructive technique of reverse Hill-Sachs defect using autologous fibular grafting.

**Methods:**

Following open reduction of the dislocated humeral head, reverse Hill-Sachs defect was reconstructed using 3–4 autologous fibular pieces (each is of 10 mm in length) fixed in flush with the articular cartilage using 4-mm cancellous screws. Defect reconstruction was then followed by modified McLaughlin’s transfer and posterior capsulorrhaphy.

**Results:**

Spherical contour of the humeral head and gleno-humeral range of motion were restored. Intra-operative dynamic testing of the reconstruct revealed no residual posterior gleno-humeral instability.

**Conclusion:**

Currently reported technique might offer advantages of graft availability, technical simplicity, familiarity and reproducibility, safety (i.e. no disease transmission) and bone preservation facilitating future revision management (if needed). Nevertheless, long-term outcomes of this technique should be investigated via further cohort clinical studies.

**Supplementary Information:**

The online version contains supplementary material available at 10.1186/s13018-021-02835-2.

## Introduction

Chronic locked posterior gleno-humeral (GH) dislocation is a rare challenging orthopaedic condition which occurs as a result of common (i.e. 72%) misdiagnosis of acute posterior GH dislocation on initial presentation (i.e. diagnostic trap). Related challenges might partially stem from difficulties encountered during achieving reduction of the humeral head back over the glenoid due to long-standing contracture, fibrosis and adhesions of the surrounding soft tissues [1–7].

Another challenge is to effectively restore mobility–stability balance of reduced GH joint. This might be accomplished via repair/reconstruction of concomitant bony (e.g. reverse Hill-Sachs defect) and soft tissue (reverse Bankart labral detachment) lesions [6, 8–12].

According to the literature, different techniques (of variable advantages and disadvantages and of datable outcomes) have been introduced for reconstruction of chronic large (25–50%) reverse Hill-Sachs defect in young active population, as McLaughlin’s subscapularis (SSC) tenodesis/Hawkins’ transposition of osteotomized lesser tuberosity into the defect, autologous iliac bone grafting of the defect and proximal humeral rotational osteotomy [1–3, 10, 13, 14].

More recently, osteo-chondral allograft reconstruction of this defect has been described with a main merit of effective restoration of humeral head sphericity, however, with concerns of graft availability, waiting list of the recipient, matching of the donor articular contour, cartilage viability, technical set-up and disease transmission [9, 11, 12, 15–18].

**Fig. 1:**
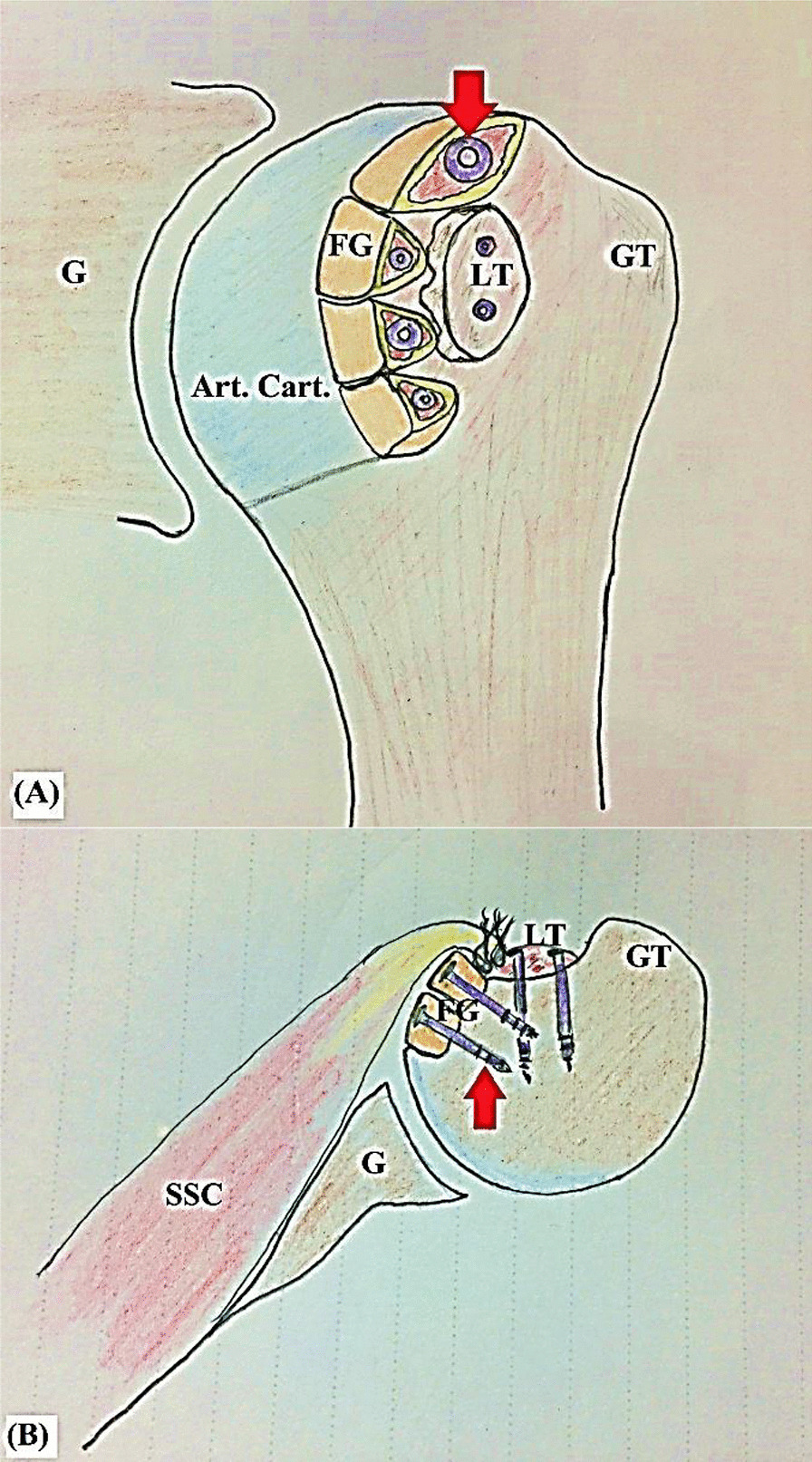
Technical principle of the currently reported reconstructive technique of reverse Hill-Sachs defect using autologous fibular grafting (fixed into the cancellous bed of the defect by red arrow-marked regular 4.0 mm cancellous screws) for restoration of spherical contour of articulating part of the humeral head in left shoulder; **A** in coronal plane; **B** in axial plane; Art. Cart., Articular cartilage of the humeral head; FG, Fibular graft pieces, G, Glenoid; GT; Greater tuberosity; LT, Lesser tuberosity; SSC, Subscapularis muscle/tendon

Based on institutional lack of allograft tissue banking, this article reports a reconstructive technique of reverse Hill-Sachs defect using autologous fibular grafting for spherical re-contouring of articulating part of the humeral head. Figure 1A, B demonstrates technical principle of the currently reported technique.

### Operative technique

The current technical note was approved by the Institutional Review Board for reconstruction of large reverse Hill-Sachs defect in patients with chronic locked posterior GH dislocation (especially in the setting of unavailability of osteo-chondral allograft).

#### 1-Set-up

**Fig. 2:**
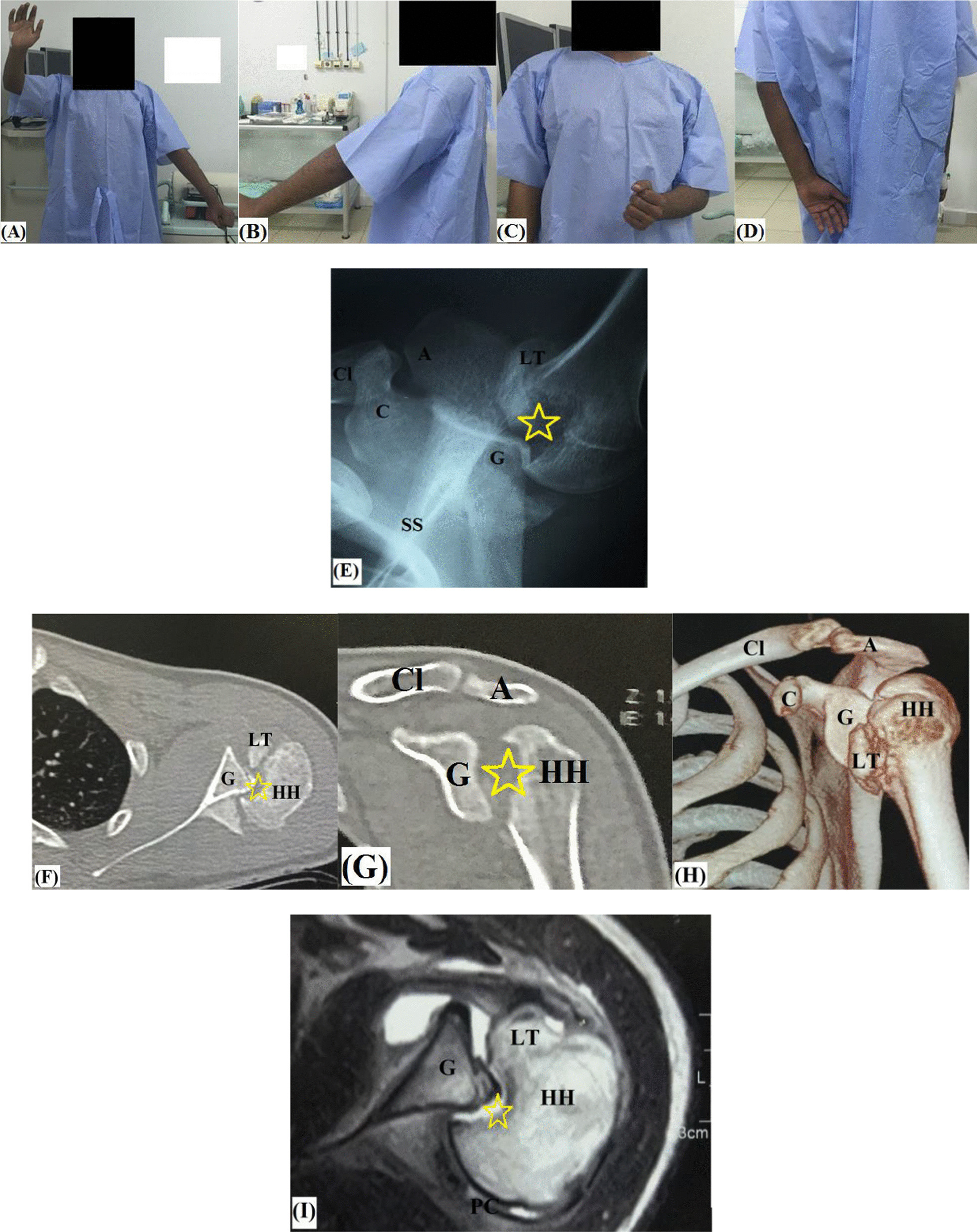
Preoperative assessment of chronic locked posterior GH dislocation (with yellow star-marked large reverse Hill-Sachs defect) in left shoulder; **A**–**D** active range of motion; **E** axillary X-ray view; **F** axial CT image; **G** coronal CT image; **H** 3D-reconstruction CT image; **I** axial MRI image; A, Acromion; C, Coracoid; Cl, Clavicle (lateral end); G, Glenoid; HH, Humeral head; LT, Lesser tuberosity; PC, Posterior capsule; SS, Scapular spine

**Fig. 3:**
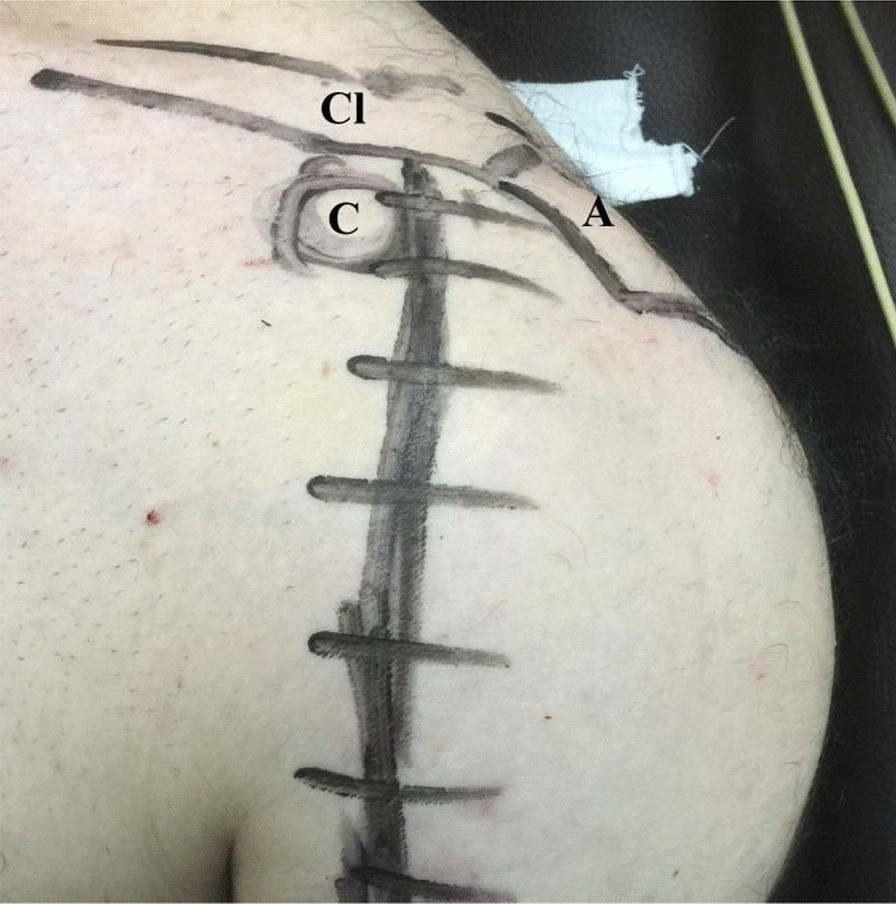
Marked-related bony and soft tissue anatomic landmarks for currently reported reconstructive technique of reverse Hill-Sachs defect in left shoulder; A, Acromion; C, Coracoid; Cl, Clavicle (lateral end)

On-theatre thorough review of clinical data and imaging studies of the patient is routinely performed. Figure 2A–I demonstrates preoperative evaluation of active range of motion and imaging modalities in chronic locked posterior GH dislocation. Following general anaesthesia and prophylactic antibiotic administration, patient is seated in beach-chair position and examined for passive range of motion (ROM) of the operated shoulder. Figure 3 demonstrates pen-marked-related bony and soft tissue anatomic landmarks.

#### 2-Surgical approach

Through standard delto-pectoral approach, subcutaneous tissue is dissected, delto-pectoral groove is defined, and cephalic vein is identified and medially retracted following cauterization/ligation of its tributaries. Sub-deltoid adhesions are released using blunt dissection to facilitate placement of sub-deltoid Homman’s retractors.

**Fig. 4:**
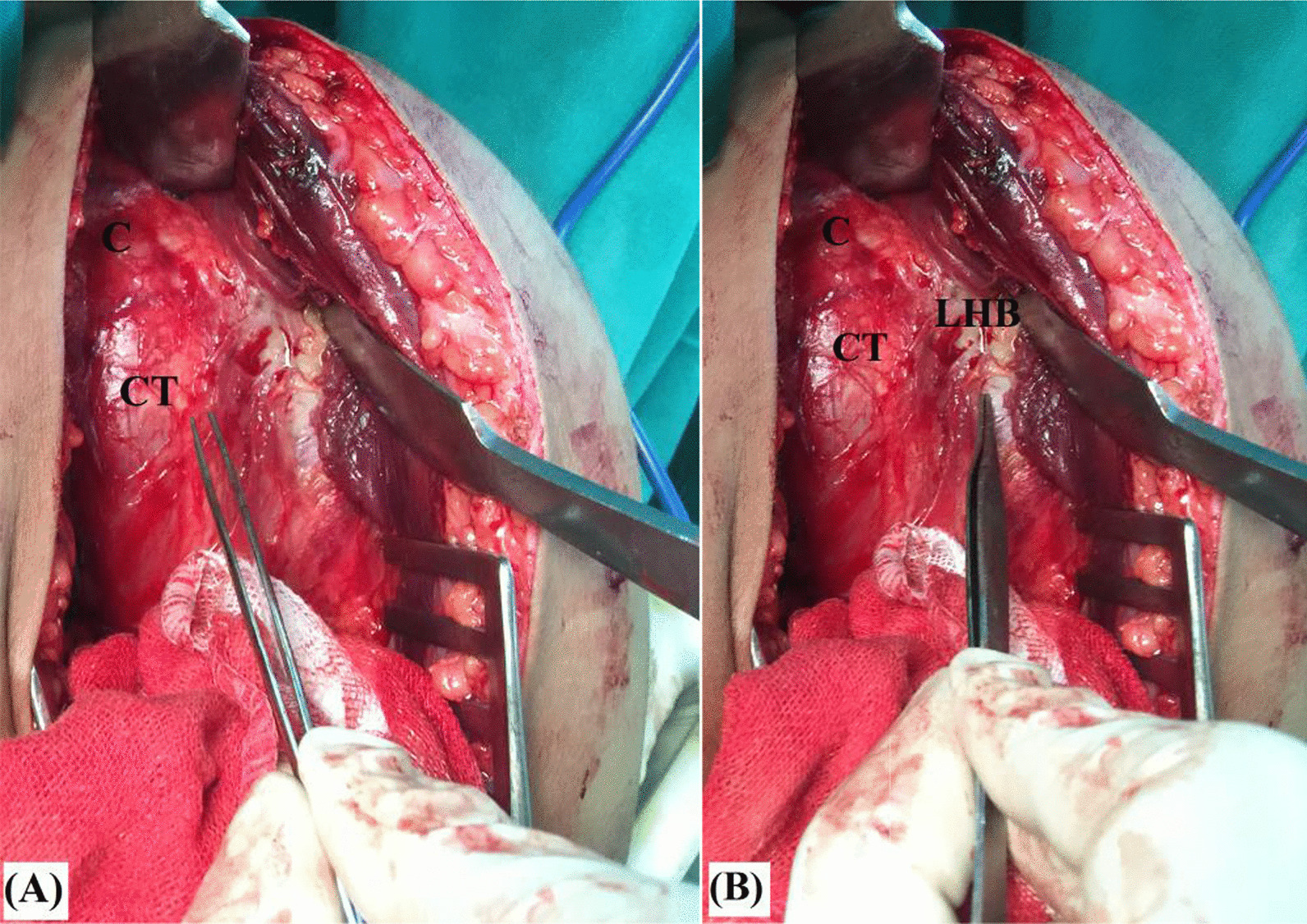
Identification of **A** the conjoint tendon; and **B** long head of biceps tendon in left shoulder; the latter is to be used as a guide for identification of the lesser tuberosity and subscapularis tendon; C, Coracoid process; CT, Conjoint tendon; LHB; Long head of biceps tendon

Due to long-standing extensive fibrosis and adhesion encountered during dissection and disturbed anatomy of locked posteriorly dislocated GH joint, coracoid process is used as cornerstone reference point during surgical approach. From this bony landmark, the conjoint tendon is identified, released from deltoid undersurface and used as a guide for recognition, release and suture-marking of long head of biceps (LHB) tendon. Figure 4A, B demonstrates identification of the conjoint and long head of biceps tendons.

Afterwards, LHB tendon (prior to its tenotomy) is used as a guide for identification of the lesser tuberosity and SSC. Overlying adhesions are released using a combination of blunt (gauze sponge) and sharp (knife blade/scissor/diathermy cauterization) dissection till full exposure of SSC and its bony insertion. Then, upper 2/3 of SSC/GH capsule (with a bony fleck) are peeled from the lesser tuberosity and medially reflected to maximize intra-articular visualization, enable GH examination (for concurrent chondral, labral and cuff lesions) and facilitate access/manoeuvres. Medial reflection of SSC is further maximized by rotator interval release. During SSC release and reflection, axillary nerve should be protected by an inferiorly placed Homman’s retractor.

#### 3-Open GH reduction

Thereafter, intra-articular adhesions are debrided exposing the plane between the humeral head (reverse Hill-Sachs defect) and the posterior glenoid. Using a sharp osteotome and mallet, this plane is gently and gradually opened to avoid unnecessary bone loss till completely freeing the humeral head from the posterior glenoid. Through this plane, a blunt-tipped broad slightly curved Homman’s retractor is introduced to lever the humeral head over the glenoid.

**Fig. 5:**
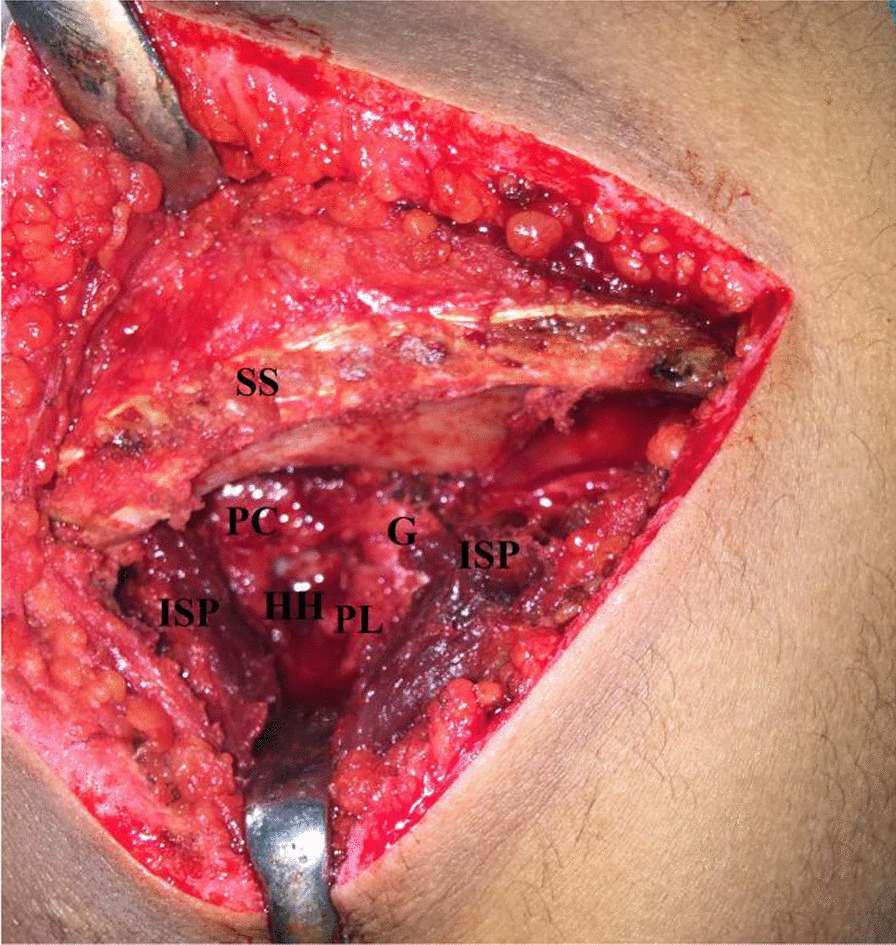
Posterior gleno-humeral capsulotomy to facilitate reduction of locked posteriorly dislocated humeral head back over the glenoid in left shoulder; G, Glenoid; HH, Humeral head; ISP, Infraspinatus muscle; PC, Posterior capsule, PL, Posterior labrum; SS, Scapular spine

In more chronic cases, GH reduction might require additional soft tissue release as detachment of upper border of pectoralis major. Alternatively, through posterior GH approach (via supraspinatus/infraspinatus or infraspinatus/teres minor interval), posterior capsulotomy and debridement/release of intervening scar tissue (between humeral head/glenoid/capsule) are performed to facilitate GH reduction. Figure 5 demonstrates posterior GH capsulotomy to facilitate reduction of locked posteriorly dislocated humeral head back over the glenoid.

#### 4-Reconstruction of reverse Hill-Sachs defect

**Fig. 6:**
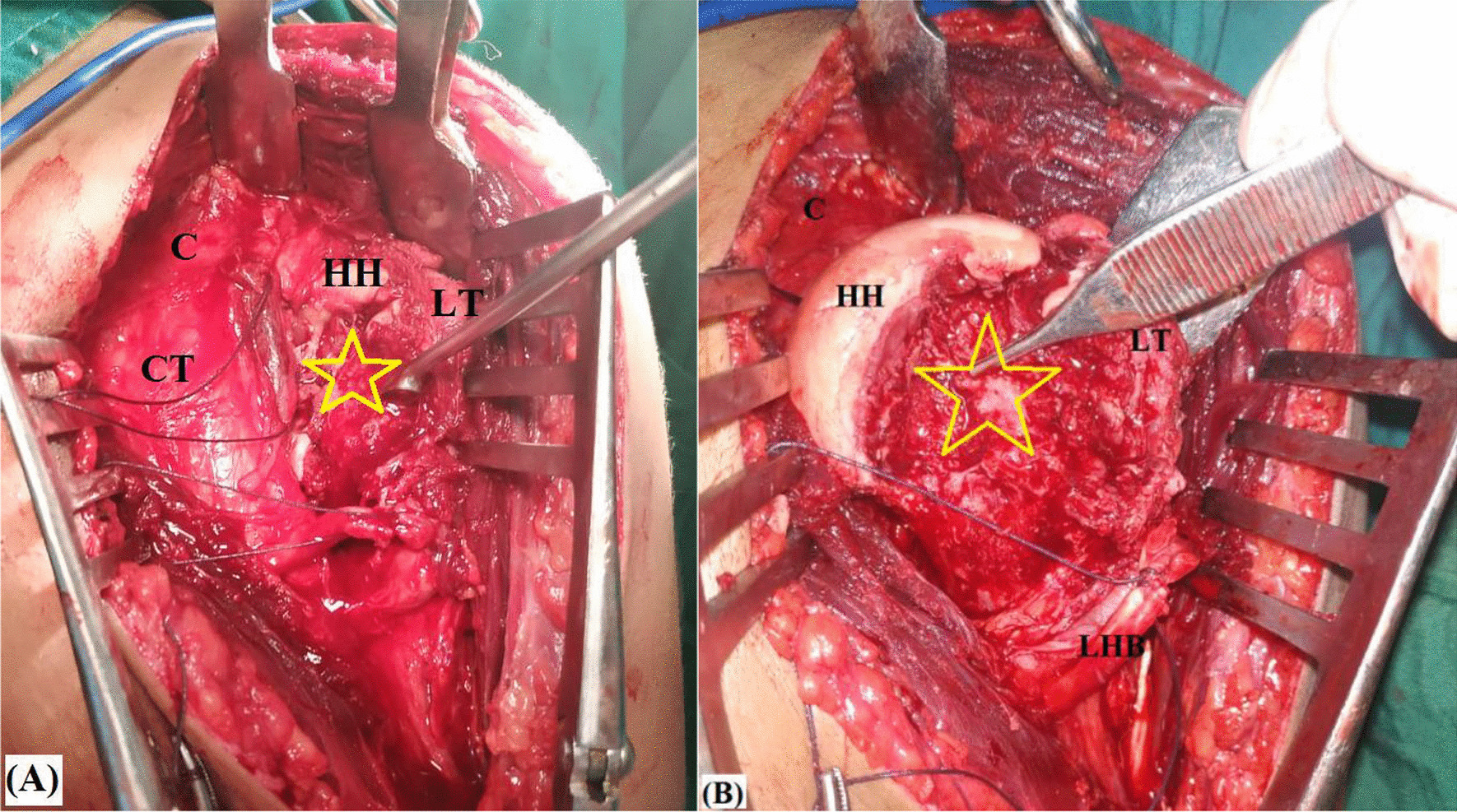
**A** Curettage; **B** microfracture of (yellow star-marked) reverse Hill-Sachs defect to improve local biology of the recipient defect for fibular graft healing in left shoulder; C, Coracoid process; CT, Conjoint tendon; HH, Humeral head; LHB; Long head of biceps tendon; LT, Lesser tuberosity

Following GH reduction, the arm is first placed in maximal external rotation for assessment of location and geometry, and for debridement, curettage and microfracture of the defect. Figure 6A, B demonstrates curettage and microfracture of reverse Hill-Sachs defect.

Next, GH joint is dynamically tested in different combinations of adduction, flexion and internal rotation (i.e. provocative position for posterior dislocation) for evaluation of defect engagement over the posterior glenoid rim and for precise planning of defect reconstruction.

Through a 5–6-cm vertical skin incision centred over middle third of lateral aspect of the leg, subcutaneous tissue is dissected, peroneal tendons are identified and posteriorly retracted, and fibular periosteal sleeve is incised and gently stripped exposing about 4–5 cm of the fibula. Based on size of reverse Hill-Sachs defect, a fibular graft of 4–5 cm in length is harvested using a combination of oscillating saw, and sharp osteotome/mallet while protecting the retracted surrounding soft tissues.

**Fig. 7:**
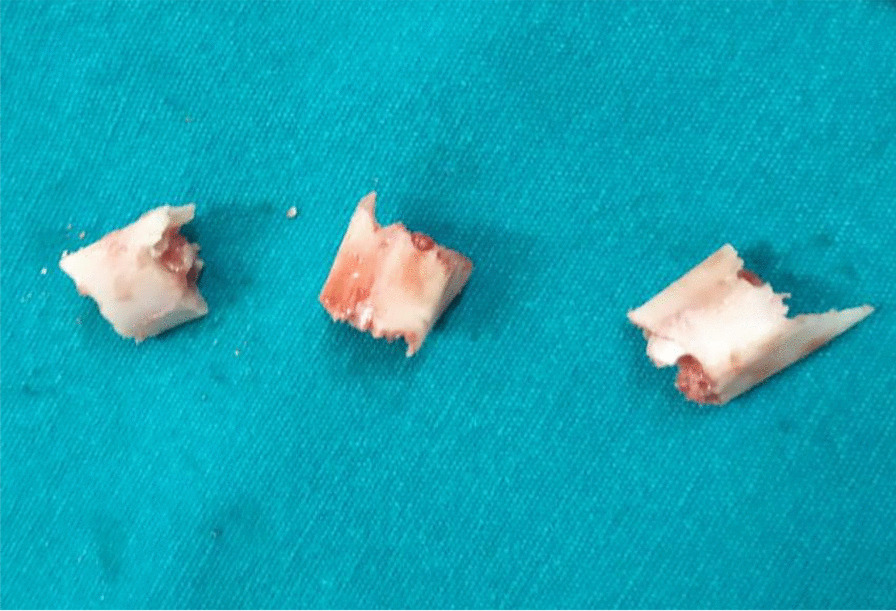
Osteotomized pieces of the harvested fibular graft before implantation into the reverse Hill-Sachs defect

Afterwards, harvested graft is stripped off soft tissue by sharp knife blade and sequentially marked at lengths of 0.8–1.2 cm according to size of reverse Hill-Sachs defect. Then, harvested graft is further osteotomized at the marked lengths by an oscillating saw into 3–4 pieces of an average length of 1 cm for each. Figure 7 demonstrates osteotomized pieces of the harvested fibular graft.

Fibular pieces are sequentially placed (tightly backed to each other) at posterior margin of the defect in flush and in parallel orientation with articular surface of the humeral head. Provisional articular (outward) surfaces of these pieces (during placement at the recipient defect) should be lateral surface of the native fibula as it is the smoothest fibular surface.

**Fig. 8:**
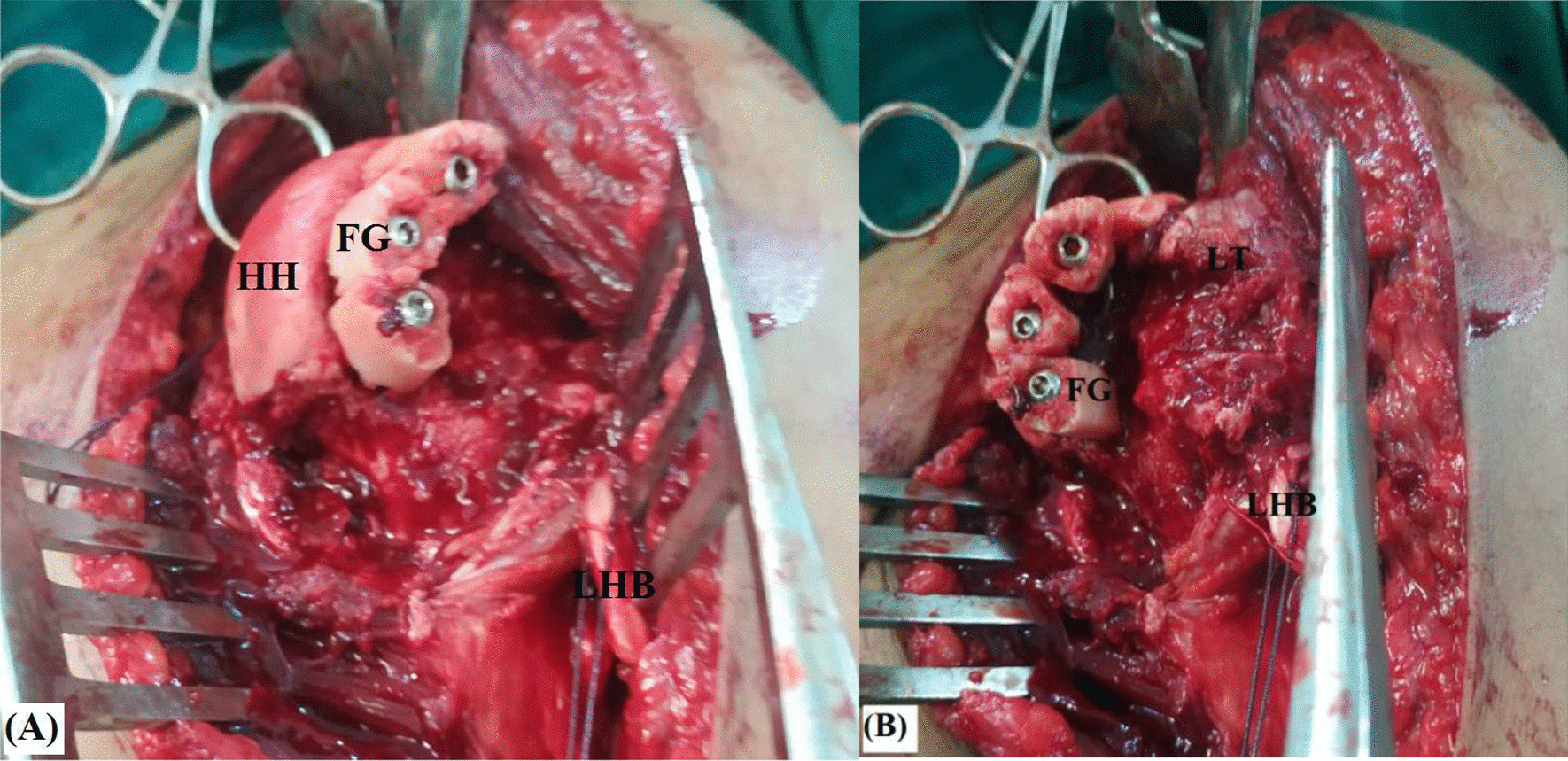
**A** Antero-medial and **B** direct anterior views of placement and fixation of fibular pieces at posterior margin of the reverse Hill-Sachs defect in left shoulder; FG, Fibular graft pieces; HH, Humeral head; LHB, Long head of biceps tendon; LT, Lesser tuberosity

Each fibular piece is fixed into the defect by a 4-mm half-threaded cancellous screw (of 30–35 mm in length) passed within medulla of the fibular piece (in countersunk fashion) towards core of the humeral head. Figure 8A, B demonstrates placement and fixation of fibular pieces at posterior margin of the reverse Hill-Sachs defect.

#### 5-Modified McLaughlin’s transfer

**Fig. 9:**
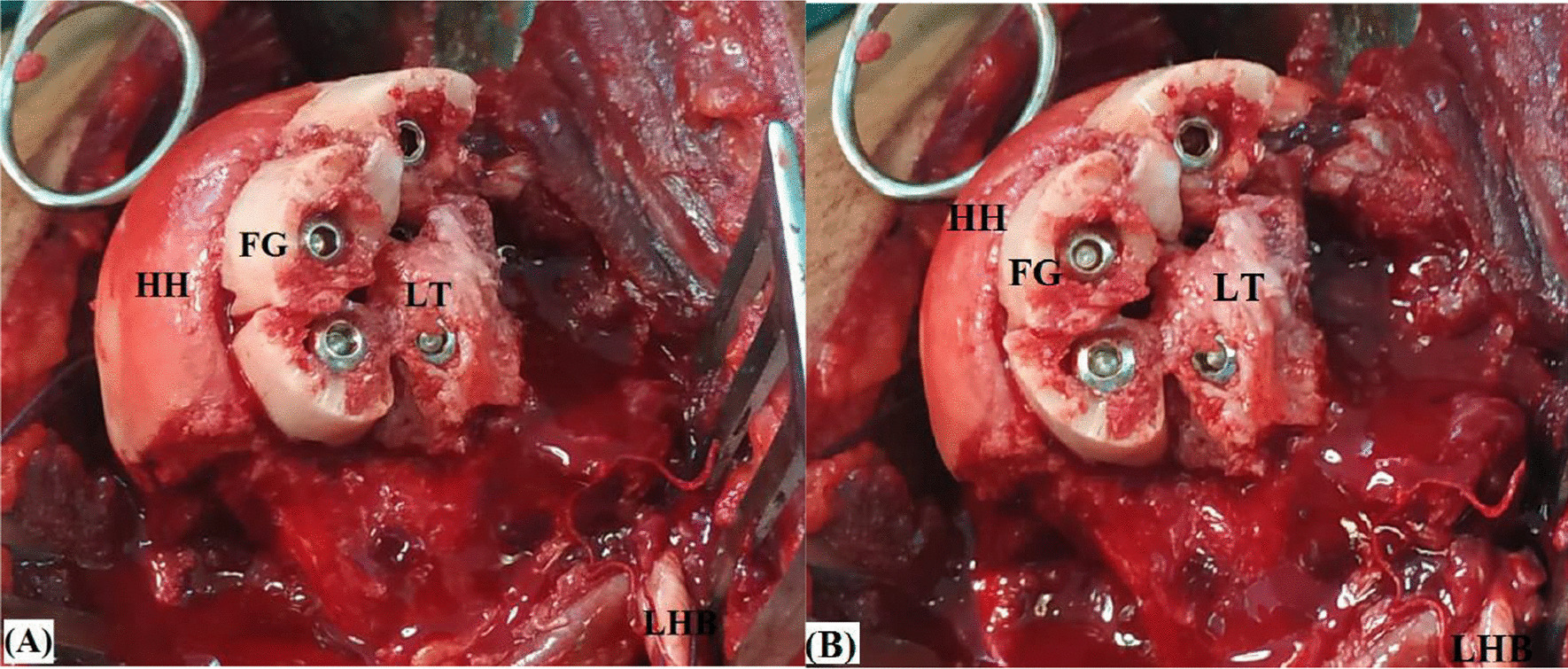
**A** Antero-medial and **B** direct anterior views of fixation of osteotomized lesser tuberosity into (anteriorly located) residual defect following fixation of the fibular graft pieces for reconstruction of reverse Hill-Sachs defect in left shoulder; FG, Fibular graft pieces; HH, Humeral head; LHB, Long head of biceps tendon; LT, Lesser tuberosity

Following fixation of the fibular pieces, (anteriorly located) residual defect is then filled by in situ bone graft from non-united fractured fragments of the proximal humerus (harvested during surgical dissection) and (when needed) by osteotomized lesser tuberosity which can be transferred and fixed to into the residual defect by one or two 4-mm half-threaded cancellous screws. Figure 9A, B demonstrates fixation of osteotomized lesser tuberosity into (anteriorly located) residual defect following fixation of the fibular graft pieces.

Thereafter, GH joint is placed in extreme provocative position for posterior dislocation to ensure that there is no longer residual reverse Hill-Sachs defect which might engage the posterior glenoid. In addition, GH joint is tested in different positions of combined abduction/adduction, flexion/extension and internal/external rotation to exclude engagement of free ends of fibular graft pieces over the anterior glenoid (otherwise, glenoid chondral damage might be a possibility) and to ascertain smooth surface joint motion of the reconstructed head over the glenoid. Pearls and pitfalls of the currently reported technique are summarized in Table 1.

**Table 1:** Pearls and pitfalls of the currently reported technique

*Pearls*
Dissection of disturbed anatomy can be facilitated by reference point of coracoid process Extensive adhesions and fibrosis can be overcome by a combination of blunt (gauze sponge) and sharp (knife blade/scissor/diathermy cauterization) dissection Tenotomy of LHB should be delayed until identification of the lesser tuberosity and SSC Release of SSC should include a bony fleck to ease future re-attachment and improve healing Medial refection of released SSC can be maximized by rotator interval release Debridement of intra-articular adhesion is essential to identify the plane between reverse Hill-Sachs defect and the posterior glenoid; this plane is gently and gradually opened by a sharp osteotome and mallet to avoid unnecessary bone loss till completely freeing the humeral head from the posterior glenoid Reduction of dislocated head can be further eased by additional release of upper pectoralis major and/or posterior GH capsulotomy Local biology for fibular graft incorporation into the defect should be enhanced by debridement, curettage and microfracture Assessment of Hill-Sachs defect in terms of size, geometry and engagement over posterior glenoid rim is crucial for appropriate intra-operative sizing and orientation of placement of fibular graft pieces For restoration of articular congruity, fibular graft pieces should be placed tightly backed to each other at the posterior margin of the defect and in flush and in parallel orientation with articular surface of the humeral head Provisional articular (outward) surfaces of these pieces (during placement at the recipient defect) should be lateral surface of the native fibula as it is the smoothest fibular surface
*Pitfalls*
Fibular graft fixation screws should be countersunk into the pieces to protect glenoid chondral surface during ROM and of appropriate length to avoid intra-articular penetration Osteotomy of lesser tuberosity should be delayed until fibular grafting of the defect and assessment of size and geometry of the residual defect Following reconstruction, GH joint should be placed in extreme provocative position for posterior dislocation to ensure that there is no longer residual reverse Hill-Sachs defect which might engage the posterior glenoid Following reconstruction, GH joint is tested in different positions to exclude engagement of free ends of fibular graft pieces over the anterior glenoid (otherwise, glenoid chondral damage might be a possibility) and to ascertain smooth surface joint motion of the reconstructed head over the glenoid Posterior GH capsulorrhaphy is essential to help centralize humeral head over the glenoid

GH, Gleno-humeral; LHB, Long Head of Biceps Brachii; ROM, range of motion; SSC, Subscapularis

#### 6-Soft tissue repair

Afterwards, SSC is trans-osseously sutured into the transferred lesser tuberosity. Tenotomized LHB tendon is managed by soft tissue tenodesis to SSC. Posteriorly, reverse Bankart repair (if needed) and posterior capsulorrhaphy of the released redundant GH capsule are performed.

#### 7-Dynamic testing of the whole construct

Prior to closure, GH joint is eventually examined for ROM and dynamic multi-directional stability. For more clarification, technical steps of the currently reported technique are illustrated in Additional file 1: Video S1.

#### 8-Postoperative rehabilitation

**Fig. 10:**
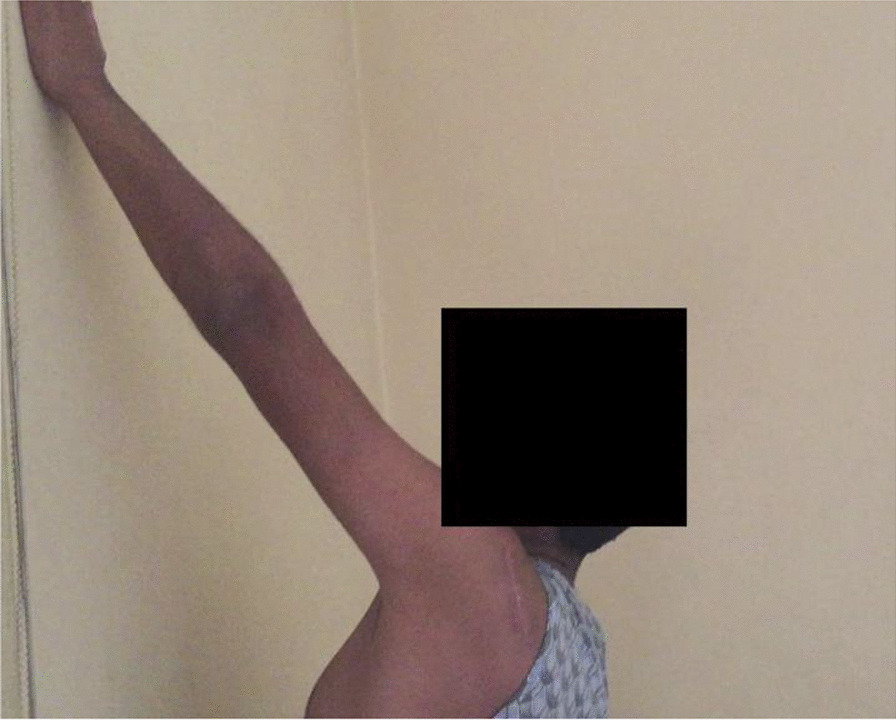
2.5-month postoperative assisted-active forward elevation of left shoulder in which sphericity of the humeral head was restored via reconstruction of large reverse Hill-Sachs defect by autologous fibular grafting

Operated shoulder is placed in a standard shoulder immobilizer for 8 weeks during which isometric deltoid exercises are encouraged. Thereafter, patient is instructed to perform at-home pendulum and assisted-active forward elevation, abduction and external/internal rotation exercises for 4 weeks. By 12 weeks postoperatively, patient is sent for physiotherapy program of 4 weeks of passive (stretching) exercises followed by 4–8 weeks of active (strengthening) exercises. Return to heavy duty and overhead activities are allowed by 20–24 weeks postoperatively. Figure 10 demonstrates 2.5-month postoperative assisted-active forward elevation of the operated shoulder.

## Discussion

On availability, reconstruction of reverse Hill-Sachs defect using osteo-chondral allograft represents an attractive management option notably in young active population. This might be attributed to its advantages of anatomic reconstruction (i.e. restoration of spherical contour and smooth articulating surface) of the humeral head, which in turn is to offer satisfactory outcomes in terms of re-establishment of GH stability/mobility balance and minimized risk of GH degenerative changes. Nevertheless, this reconstructive option has disadvantages of disease transmission, limited worldwide availability of tissue banking, relatively high non-union rates, graft resorption and inferior biomechanical properties. To overcome the latter disadvantages, the current technical note describes a reconstructive technique of reverse Hill-Sachs defect using fashioned autologous fibular graft [9, 11, 12, 15–18].

The current technique *is primarily indicated for spherical re-contouring of the humeral head in* patients of chronic locked posterior GH dislocation with large (i.e. 25–50%) reverse Hill-Sachs defect engaging over the posterior glenoid rim. Table 2 summarizes indications and contraindications of the currently reported technique.

**Table 2:** Indications and contraindications of the currently reported technique

*Indications*
Reconstruction of large (i.e. 25–50%) reverse Hill-Sachs defect engaging over the posterior glenoid rim in young active patients with: a-chronic locked posterior GH dislocation b-recurrent posterior GH instability c-high risk for posterior GH instability recurrence (e.g. hyper-laxity) d-revision management of posterior GH instability
*Contraindications*
Massive (> 50%) reverse Hill-Sachs lesion Arthritic GH joint Concurrent irreparable rotator cuff tears in the elderly

GH, gleno-humeral

For close reproduction of humeral head sphericity, some technical pearls of the current note should be considered. First, utmost care should be paid during placement/fixation of fibular pieces which should be kept tightly packed to each other, and levelled and in parallel orientation with intact chondral surface of the humeral head (to avoid step-off of the reconstructed surface). In addition, length of each fibular piece should not exceed 1–1.2 cm to keep the reconstructed surface spherical as closely as possible (i.e. for simulation of radius of curvature of the native humeral head); otherwise, longer pieces will offer obviously flattened reconstructed surface. Restoration of spherical contour can be further ascertained by moving the reconstructed head over the glenoid looking for step-off clicking or catching.

The current reconstructive technique might offer a number of technical, biomechanical and biological advantages. *From a technical perspective*, 3D-planning of the current description (via evaluation of location, geometry and posterior glenoid engagement of reverse Hill-Sachs defect) for proper placement of the fibular graft pieces is mainly an intra-operative step. Meanwhile in allograft reconstruction, additional advanced preoperative planning (by three-dimensional computed tomography [3D-CT] and modelling) is recommended. Furthermore, associated soft tissue lesions (i.e. posterior capsular redundancy and reverse Bankart lesion) should be well appreciated on MRI as well [11, 12, 19, 20].

Half-threaded screws were implants of choice due to their familiar use in orthopedic surgical practice and to ensure sufficient compression at fibular graft-cancellous bed interface. In addition, medullary geometry of the fibular pieces allows these screws to be inserted in countersunk fashion. However, intra-articular screw penetration should be ruled out by direct GH inspection.

Meanwhile, fixation methods of allograft (e.g., headless cannulated screws/press-fit techniques) are more technically-demanding when compared with regular screws. And in a comprehensive view, it isn’t unjust to mention that defect reconstruction using osteo-chondral allograft is somewhat a complex procedure as it necessitates tissue banking facilities; and as well, special instrumental setup and measurements for fashioning the recipient defect and the graft into reciprocal orange-slice configuration [9, 12, 18].

On the contrary, the current technical description owns the advantages of graft availability and technical simplicity as it does not require special set-up, instrumentations, measurements (for graft size matching) or graft preparation. In addition, most of orthopaedic shoulder surgeons are familiar with its major technical bulk (e.g. fibular graft harvesting, preparation and fixation). A technical comparison of the currently reported technique versus its counterpart of osteo-chondral allograft is summarized in Table 3.

**Table 3:** Technical differences of the currently reported technique from that of osteo-chondral allograft

Technical difference	Current technique	Osteo-chondral allograft
Approach	Open	Open
Measurement of defect size	–	Essential
Reconstructive graft	Autologous fibula	Osteo-chondral allograft (humeral head, femoral head, talus)
Graft availability	Readily available	Necessity of tissue banking systems
Graft preparation and fashioning	Simplified	Technically demanding
Graft bone marrow elements	–	Washed out by copious lavage
Fixation method	4 mm Cancellous screws	Press-fit Countersunk screws/pins
Enhancement of local biology	Microfracture	Platelet-rich plasma
Concurrent posterior GH capsulorrhaphy and McLaughlin’s procedures	Feasible	Feasible
Reconstructed articular surface	Lateral surface of native fibula + Fibrocartilage	Articular cartilage

LHB, Long Head of Biceps Brachii; SSC, Subscapularis

Furthermore, the current note provides an effective biological reconstructive option of the humeral head in young active population in whom (partial/total) resurfacing/replacement prosthetics are contra-indicated. Moreover, it is a bone stock-preserving procedure which does not interfere with future arthroplasty (if needed) [5, 9].

**Fig. 11:**
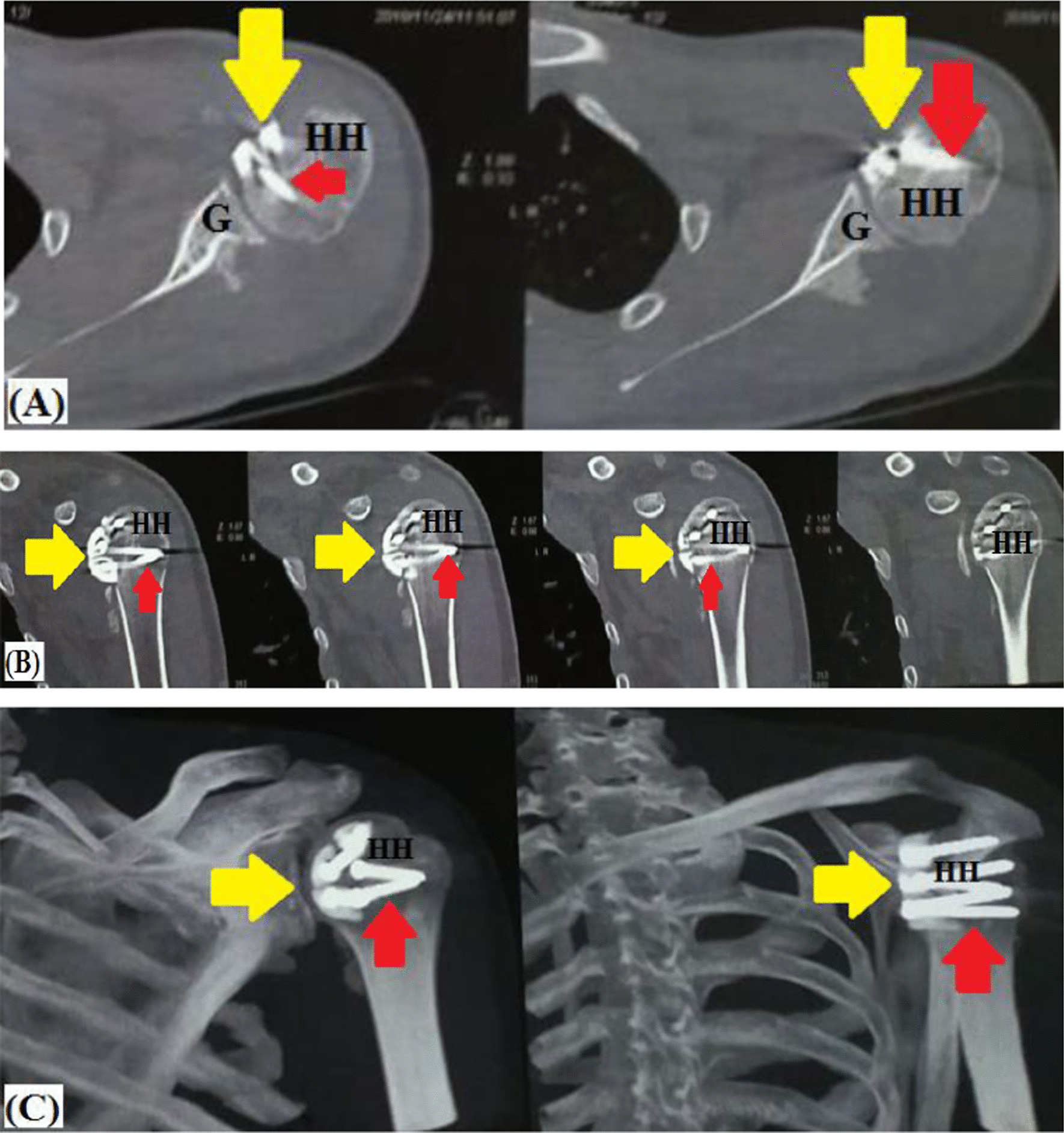
Postoperative imaging of restored humeral head sphericity and articular congruity following (red arrow-marked) screw fixation of (yellow arrow-marked) fibular graft pieces into reverse Hill-Sachs defect in left shoulder; **A** axial CT image; **B** coronal CT image; **C** 3D-reconstruction CT image; G, Glenoid; HH, Humeral head

**Fig. 12:**
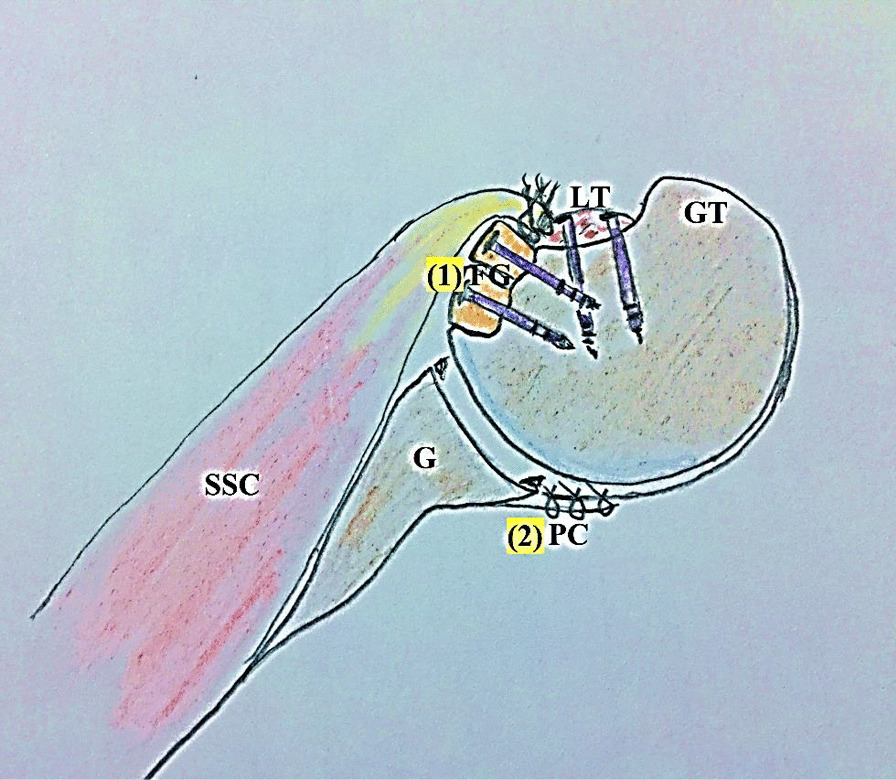
Biomechanical background of the currently reported technique in left shoulder; (1) restoration of humeral head sphericity; and as a sequel, congruent gleno-humeral articular arc is increased and engagement is negated; (2) capsulorrhaphy of the lax, redundant and stretched-out posterior gleno-humeral capsule is to help keep the humeral head centred over the glenoid; FG, Fibular graft pieces, G, Glenoid; GT; Greater tuberosity; LT, Lesser tuberosity; PC, Posterior gleno-humeral capsule; SSC, Subscapularis muscle/tendon

From a biomechanical perspective, the current technique can re-establish posterior GH stability via two static mechanisms, the first is restoration of humeral head sphericity, and as a sequel, congruent GH articular arc is increased and engagement is negated. Figure 11A–C demonstrates postoperative imaging of restored humeral head sphericity and GH articular congruity following fibular grafting of reverse Hill-Sachs defect. Meanwhile, the second mechanism of capsulorrhaphy of the lax, redundant and stretched-out posterior GH capsule helps keep the humeral head centred over the glenoid. Figure 12 demonstrates biomechanical background of the currently reported technique.

Recommendations of posterior capsular repair/reconstruction following reconstruction of reverse Hill-Sachs defect are still inconclusive. For cases of chronic locked posterior GH dislocation, Matthewson et al. described an arthroscopic technique of posterior GH capsular augmentation using a dermal allograft as well as McLaughlin reconstruction of reverse Hill-Sachs defect, pointing out that despite closed reduction of the GH joint was feasible prior to reconstruction; however, capsular augmentation was technically demanding due to posterior GH scarring and graft tangling. In accordance, Mitchel et al. introduced an arthroscopic repair technique of posterior gleno-humeral ligament/labrum avulsion prior to reconstruction of Hill-Sachs defect using fresh talar osteo-chondral allograft, emphasizing the necessity of addressing posterior capsular lesions encountered in chronic posterior GH dislocation to avoid postoperative persistence of posterior GH instability [11, 21, 22].

In addition, another biomechanical advantage of the current description is smooth hard articular surface offered by the implanted fibula; which might have superior biomechanical properties than its allograft counterpart and might more closely resemble those of metallic partial articular resurfacing implants. A biomechanical comparison of the currently reported technique versus its counterpart of osteo-chondral allograft is summarized in Table 4.

**Table 4:** A comparison of gleno-humeral re-stabilization mechanisms of the currently reported technique versus its counterpart of osteo-chondral allograft

Mechanism	Reported technique	Osteo-chondral allograft
Restoration of humeral head sphericity and smooth articular arc	+	+
Concurrent posterior capsulorrhaphy	±	±
Conversion of intra-articular residual reverse Hill-Sachs defect into an extra-articular one by lesser tuberosity (± SSC) trans-fixation into the defect	Might be needed following fibular grafting according to intra-operative assessment of geometry and engagement of the residual defect	Not needed as it fully reconstructs the defect

SSC, Subscapularis

**Fig. 13:**
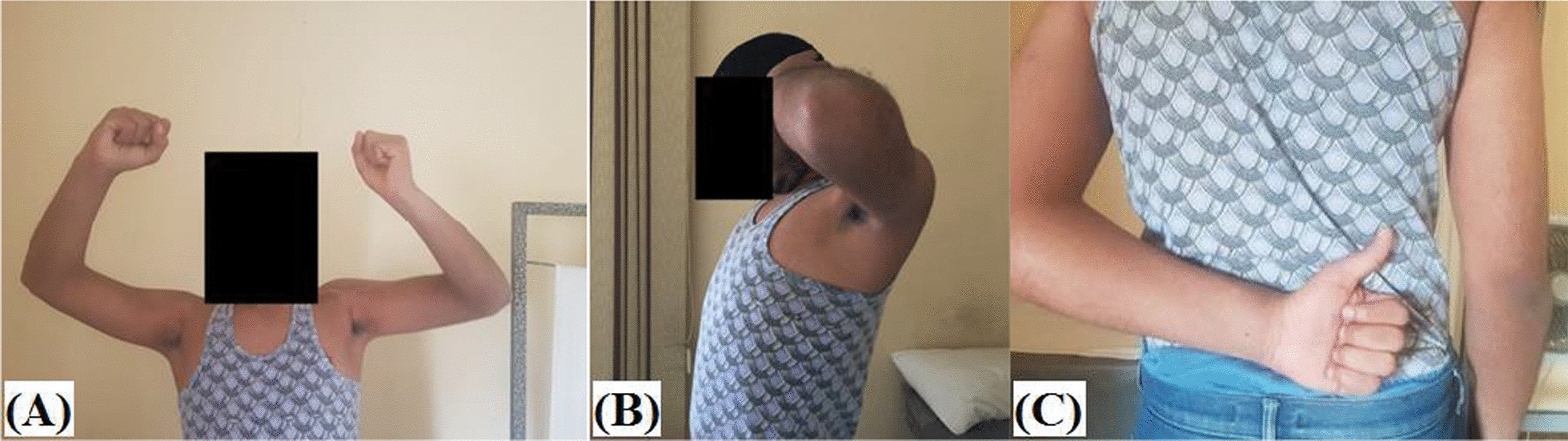
2.5-month postoperative range of motion of reconstructed left shoulder by fibular grafting of reverse Hill-Sachs defect

Furthermore, the current technique does not limit range of external rotation of the reconstructed GH joint as it per se does not necessitate lesser tuberosity transposition/SSC tenodesis into reverse Hill-Sachs defect, and when the latter are concurrently needed, transposition/tenodesis are performed into a less medialized position. Figure 13A–C demonstrates a postoperative range of motion of reconstructed shoulder by fibular grafting of reverse Hill-Sachs defect. The former remark might be supported by reported outcomes (i.e., postoperative insignificant ROM deficits and significantly improved pain and functional scores) of two different series of modified Hawkins’ techniques of Shams et al. (i.e. re-attachment of the transferred lesser tuberosity by non-absorbable sutures in 11 patients) and of Arafa et al. (i.e. dual SSC procedure of concurrent lesser tuberosity transfer and residual defect filling with a part of SSC tendon in 12 patients) [10, 23].

*As regards graft biology,* a major concern of allograft reconstruction is bone marrow elements which should be intra-operatively washed out prior to graft fixation using copious lavage for at least 5 min. In addition, allograft-soaking in autologous plasma/platelet-rich plasma is recommended to overcome its relatively low healing rate [12].

**Fig. 14:**
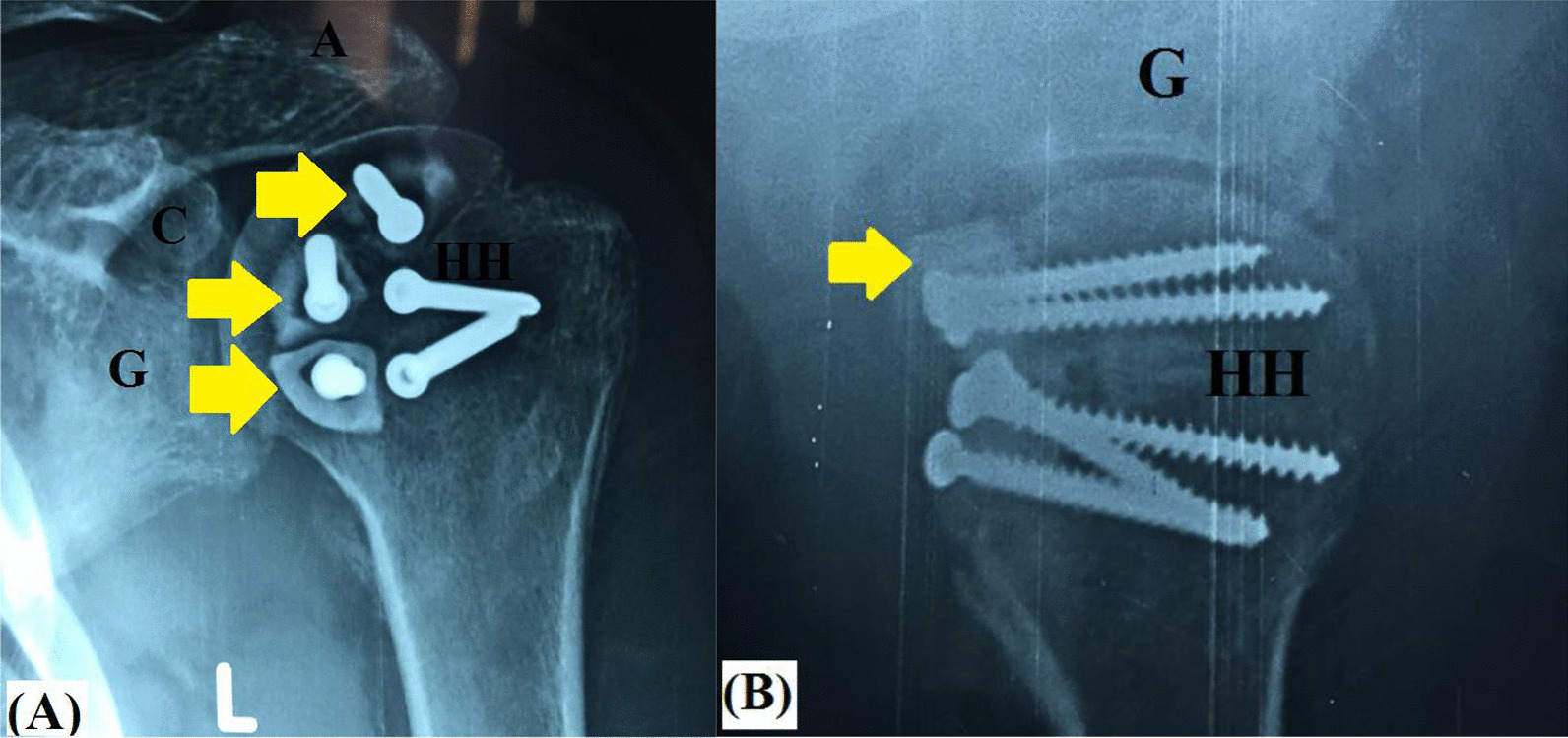
2.5-month postoperative ongoing healing of the (yellow arrow-marked) fibular graft into cancellous bed of reverse Hill-Sachs defect in left shoulder; **A** antero-posterior X-ray view; **B** axillary X-ray view; A, Acromion; C, Coracoid; G, Glenoid; HH, Humeral head

Meanwhile in the current technical note, fibular graft incorporation can be accelerated by enhancement of the local biology via debridement and microfracture of reverse Hill-Sachs defect. Besides, microfracture might stimulate fibrocartilage formation over the cancellous bed of the defect and possibly to lesser extent over the fibular graft itself. In addition, the reported technique employs small-sized pieces of non-vascularized fibular graft which have well-established high rate of graft healing/incorporation. Figure 14A, B demonstrates ongoing healing of the fibular graft into cancellous bed of reverse Hill-Sachs defect.

On the other hand, *a technical limitation* of the current description is that it does not fully reconstruct the humeral head notably in large reverse Hill-Sachs defect. Nevertheless, it is still able to convert engaging defect into non-engaging one, so that GH mobility/stability balance is restored. According to Black et al., reverse Hill-Sachs defect can be partially reconstructed using osteo-chondral allograft as long as any residual defect is not large enough to engage the posterior glenoid rim. An option to overcome the previous limitation is to couple the current technique with osteotomy and transposition of the lesser tuberosity (± SSC tenodesis) into the anteriorly located residual defect in order to fill the latter or convert the latter into an extra-articular one [9, 12, 23].

What also raises concerns is possibility of engagement of free (anterior) ends of the fibular pieces over the (anterior) glenoid rim at terminal range of internal rotation. The preceding possibility can be ruled out by repeated intra-operative evaluation of GH motion in different provocative positions. And if this anterior engagement is still questionable, the current technique can be coupled with Hawkins’s transposition of the lesser tuberosity with SSC tenodesis into the fibular graft.

It is worth mentioning that long-term functional outcomes of the current technical note should be validated via further clinical studies. The latter might also include use of different imaging modalities (e.g. CT, MRI) for more accurate assessment of fibular graft incorporation into the recipient humeral defect. Advantages and disadvantages of the currently reported technique are summarized in Table 5.

**Table 5:** Advantages and disadvantages of the currently reported technique

*Advantages*
Restoration of GH smooth articulating surfaces and articular congruity Graft availability Technical simplicity, familiarity, quickness, safety and reproducibility Feasible concurrent procedures (e.g. posterior capsulorrhaphy, McLaughlin’s/Hawkins’s procedures) In situ enhancement of local biology for graft incorporation Cost-saving (regular screws) Bone-preserving procedures; no interference with future arthroplasty Avoidance of osteo-chondral allograft-related drawbacks (e.g. disease transmission, unavailability of tissue banking, non-union, bone marrow elements and biomechanical properties) No marked loss of GH range of motion (i.e. external rotation) Relatively easy revision
*Limitations*
Non-anatomic reconstruction of GH articulating surfaces Technical irreproducibility in extensive defects Incomplete reconstruction of reverse Hill-Sachs defect Possible need of concurrent McLaughlin’s/Hawkins’s procedures Donor site morbidity No biomechanical validation No long-term cohort clinical studies

GH, Gleno-humeral

## Conclusion

In the setting of osteo-chondral allograft unavailability, current technical description of autologous fibular grafting of reverse Hill-Sachs defect might restore smooth congruent articulating surface of the humeral head in patients with chronic locked posterior gleno-humeral dislocation. It may herald advantages of technical simplicity, familiarity and reproducibility as well. However, further clinical studies are needed to validate its long-term outcomes.

## Supplementary Information

**Additional file 1**. **Video S1** Demonstrate technical principle of the currently reported reconstructive technique of reverse Hill-Sachs defect using autologous fibular grafting for restoration of spherical contour of articulating part of the humeral head in left shoulder following seating the patient in beach-chair position and pen-marking of related bony and soft tissue anatomic landmarks. **1-** Through delto-pectoral approach, the coracoid process, the conjoint tendon and long head of biceps (LHB) tendon are identified. Overlying adhesions are released till full exposure of subscapularis (SSC) and its bony insertion. **2-** Upper 2/3 of SSC/gleno-humeral (GH) capsule (with a bony fleck) is peeled from the lesser tuberosity and medially reflected to maximize intra-articular examination (for concurrent chondral, labral and cuff lesions). **3-** Medial reflection of SSC is further maximized by rotator interval release. **4-** Thereafter, intra-articular adhesions are debrided exposing the plane between the humeral head (reverse Hill-Sachs defect) and the posterior glenoid. **5-** Using a sharp osteotome and mallet, this plane is gently and gradually opened till completely freeing the humeral head from the posterior glenoid. Through this plane, a blunt and broad slightly curved Homman’s retractor is introduced to lever the humeral head over the glenoid. **6-** In more chronic cases, GH reduction might require additional soft tissue release as detachment of upper border of pectoralis major. Alternatively, through posterior GH approach (via supraspinatus/infraspinatus or infraspinatus/teres minor interval), posterior capsulotomy and debridement/release of intervening scar tissue (between humeral head/glenoid/capsule) are performed to facilitate GH reduction. **7-** Then, the arm is first placed in maximal external rotation for assessment of location and geometry, and for debridement, curettage and microfracture of the reverse Hill-Sachs defect in order to improve local biology of the recipient defect for graft healing. **8-** Next, GH joint is placed in provocative position for posterior dislocation for evaluation of defect engagement on the posterior glenoid rim and for precise planning of defect reconstruction. **9-** Through a vertical skin incision over lateral aspect of the fibula, subcutaneous tissue is dissected, peroneal tendons are identified and posteriorly retracted, and fibular periosteal sleeve is incised and gently stripped exposing about 4–5 cm of the fibula. Based on size of reverse Hill-Sachs defect, a fibular graft of 4-5 cm in length is harvested. **10-** Harvested graft is sequentially marked at a length of 0.8–1.2 cm and further osteotomized at the marked lengths by an oscillating saw into 3–4 pieces of an average length of 1 cm for each. **11-** Fibular pieces are sequentially placed (tightly backed to each other) at posterior margin of the defect in flush and in parallel orientation with articular surface of the humeral head. Provisional articular (outward) surfaces of these pieces (during placement at the recipient defect) should be lateral surface of the native fibula as it is the smoothest fibular surface. **12-** Each fibular piece is fixed into the defect by a 4-mm half-threaded cancellous screw (of average length of 30–35 mm) passed within medulla of the fibular piece (in countersunk fashion) towards core of the humeral head. **13-** Following fixation of fibular pieces, (anteriorly located) residual defect is then filled by in situ bone graft from non-united fractured fragments of the proximal humerus (harvested during surgical dissection) and (when needed) by osteotomized lesser tuberosity which is transferred and fixed to into the residual defect by one or two 4-mm half-threaded cancellous screws. **14-** Thereafter, GH joint is placed in extreme provocative position for posterior dislocation to ensure that there is no longer residual reverse Hill-Sachs defect which might engage the posterior glenoid. In addition, GH joint is tested in different positions to exclude engagement of free ends of fibular graft pieces over the anterior glenoid (otherwise, glenoid chondral damage might be a possibility) and to ascertain smooth surface joint motion of the reconstructed head over the glenoid. **15-** Afterwards, SSC is trans-osseously sutured into transferred lesser tuberosity. Tenotomized LHB tendon is managed by soft tissue tenodesis to SSC. Posteriorly, reverse Bankart repair (if needed) and posterior capsulorrhaphy of the released redundant GH capsule are performed. **16-** Prior to closure, GH joint is eventually examined for range of motion and dynamic multi-directional stability.

## Data Availability

Materials of the current work are available as a supplementary file (video form).

## Abbreviations

GH: Gleno-humeral
LHB: Long head of biceps
ROM: Range of motion
SSC: Subscapularis
3D-CT: Three-dimensional computed tomography
